# Acute Myelogenous Leukemia Cells Secrete Factors that Stimulate Cellular LDL Uptake via Autocrine and Paracrine Mechanisms

**DOI:** 10.1007/s11745-017-4256-z

**Published:** 2017-05-09

**Authors:** Hasanuzzaman Bhuiyan, Michèle Masquelier, Loukas Tatidis, Astrid Gruber, Christer Paul, Sigurd Vitols

**Affiliations:** 1 Department of Medicine, Clinical Pharmacology Unit, Karolinska Institute, Karolinska University Hospital, Solna, 17176 Stockholm, Sweden; 2 Centre for Haematology and Regenerative Medicine, Karolinska Institute, Karolinska University Hospital, Solna, 171 76 Stockholm, Sweden; 3 Centre for Haematology and Regenerative Medicine, Karolinska Institute, Karolinska University Hospital, Huddinge, 141 86 Stockholm, Sweden

**Keywords:** Acute myelogenous leukemia, Cholesterol, Low density lipoprotein receptor, Cytokines

## Abstract

Leukemic cells isolated from most patients with acute myelogenous leukemia (AML) have higher low density lipoprotein (LDL) uptake than normal mononuclear blood cells. Little is known, however, about the mechanism behind the elevated LDL uptake. We investigated if AML cells secrete factors that stimulate cellular LDL uptake. Mononuclear blood cells were isolated from peripheral blood from 42 patients with AML at diagnosis. Cellular LDL uptake was determined from the degradation rate of ^125^I-labelled LDL. Conditioned media from AML cells stimulated the LDL degradation in the leukemic cell lines KG1 and HL60, and in isolated AML cells. The stimulatory effect correlated with the LDL degradation in the AML cells directly after isolation from blood. Conditioned media also autostimulated LDL degradation in the AML cells themselves. Concentrations of IL-6 and IL-8 in AML cell conditioned media correlated with the LDL degradation in AML cells directly after isolation from blood. Addition of R-TNF-α, but not IL-6 or IL-8, stimulated LDL degradation in HL60, KG1, and AML cells. The LDL degradation in AML cells could be inhibited by a LDL receptor blocking antibody. AML cells secrete factors that stimulate LDL uptake in a paracrine and autocrine pattern which open up therapeutic possibilities to inhibit the uptake of LDL by administration of antibodies to these factors.

## Introduction

Human cells have receptors for low density lipoprotein (LDL), the major cholesterol-carrying lipoprotein in human plasma. Leukemic cells from most patients with acute myelogenous leukemia (AML) have an elevated high affinity degradation rate of LDL [1, 2]. The cellular degradation rate of LDL is an indirect measure of the total cellular uptake of LDL since the degradation products that are released into the incubation medium reflects total cellular uptake with time. The activity of HMG-CoA reductase, the rate limiting enzyme for endogenous cholesterol synthesis [3, 4], is also elevated in leukemic cells from AML patients [2, 5]. The expressions of LDL receptors as well as HMG-CoA reductase are normally regulated by the intracellular cholesterol level [6] through end-product repression. The mechanism underlying the abnormal cholesterol homeostasis in AML cells is unknown. A lower cholesterol content has been reported for AML cells compared to normal mononuclear blood cells [7–10] supporting the hypothesis that the elevated LDL uptake is a compensatory response to a low cellular cholesterol. However, we have previously shown that there is no correlation between the cellular cholesterol content of AML cells and the LDL degradation rate [10].

We earlier found a decreased feedback regulation of LDL uptake by sterols in AML cells [10]. Both sterol resistance and the white blood cell count in AML patients correlated with the LDL degradation rate in AML cells directly after isolation from blood [10], indicating that a growth related and sterol unresponsive mechanism influences the LDL uptake. It is known that AML cells synthesize and secrete cytokines and growth factors [11, 12]. Previous studies have shown that conditioned medium from gall bladder cancer cells stimulated the LDL degradation rate in human skin fibroblasts [13] and conditioned medium from porcine smooth muscle cells stimulated the LDL degradation rate in HepG2 cells [14]. The hypothesis that autocrine/paracrine factors play a role in regulating LDL uptake is further supported by observations made by Grove *et al.* that oncostatin M (OSM), secreted by macrophages, increases LDL uptake in HepG2 cells [15]. This led further to the identification of a novel sterol-independent regulatory element in the LDL receptor promoter that mediates OSM induced transcription of the LDL receptor gene [16, 17]. These findings illustrate the complexity of cellular receptor mediated LDL uptake regulation and are also in agreement with our observations that AML cells have decreased feedback regulation of LDL uptake by sterols [5, 10].

Considering that incubation of cells with cytokines and mitogenic substances have been shown to stimulate LDL receptor gene expression and cause sterol resistance [13, 15, 18–22], we hypothesized that leukemic cells from AML patients synthesize cytokines/growth factors that autostimulate LDL uptake and cause decreased responsiveness to sterols.

We therefore investigated if media conditioned by AML cells stimulate LDL degradation in the myeloid cell lines KG1 and HL60, and in the isolated AML cells themselves. We also measured the concentration of several putative cytokines (IL-1β, IL-2, IL-4, IL-6, IL-8, IL-18, IFN-γ and TNF-α) and growth factors (vascular endothelial growth factor, VEGF, hepatocyte growth factor, HGF and, basic fibroblast growth factor, bFGF) in AML cell conditioned media and studied the effects of adding recombinant cytokines and neutralizing antibodies on cellular LDL degradation.

## Materials and Methods

### Lipoproteins

LDL (density 1.020–1.063 g/mL) and human lipoprotein deficient serum (LPDS; density >1.215 g/mL) were isolated from serum of healthy blood donors by sequential ultracentrifugation [23]. The purity of LDL and LPDS preparations was examined by agarose gel electrophoresis, and the absence of cholesterol in LPDS was confirmed by enzymatic cholesterol analysis (Merck, Darmstadt, FRG). Na^125^I (IMS 30) was obtained from Amersham (Little Chalfont, UK). ^125^I-labeled LDL (specific activity, 130–375 cpm/ng protein) was prepared as described by Langer *et al.* [24]. Less than 1% of the radioactivity in the ^125^I-LDL preparations was present as free iodide. The concentration of LDL refers to protein and was determined using bovine serum albumin as the standard [25].

### Blood Collection and Cell Isolation Procedure

Heparinized peripheral blood samples were obtained from consecutive patients at diagnosis. (Table 1) and healthy blood donors at Karolinska university hospital. AML was classified according to the French-American-British (FAB) sub-classification system [26]. Mononuclear blood cells were isolated from blood by centrifugation at 4 °C on Lymphoprep (density 1.077 g/mL) (Nycomed Pharma AS, Oslo, Norway), [27] and washed three times with ice cold PBS. Cell number was determined using a Coulter counter Z2 (Beckman Coulter, Fullerton, CA, USA). The study was approved by the regional ethical committee in Stockholm and informed consent was obtained from all subjects.

**Table 1:** Characteristics of AML patients studied

Number	Age (years) mean (range)	White blood cell count (10^6^/mL) mean (range)	Basal high affinity LDL degradation rate ng/h/10^6^ cells mean (range)
42	61 (23–93)	72 (17–278)	2.82 (0.06–8.89)

### Cell Lines and Culture Conditions

The human acute promyelocytic leukemia cell line HL60 and the human acute myeloid leukemia cell line KG1 were purchased from the American type culture collection (Manassas, VA, USA). The cells were cultured in RPMI 1640 growth medium supplemented with 10% calf serum and antibiotics (100 IU penicillin + 100 mg streptomycin/mL) in a humidified incubator at 37 °C in 25 or 75-cm^2^ tissue culture dishes (Costar Corporation, Cambridge, Ma, USA). The cells were subcultured two to three days before experiments in order to perform experiments on subconfluent dividing cells (approximate cell concentration 1 × 10^6^ cells/mL during the experiments).

### Preparation of Conditioned Medium

Conditioned medium from isolated AML cells was obtained by cultivating the cells, at a concentration of 2.5 × 10^6^ cells/mL, at 37 °C in 1640 RPMI medium supplemented with 5 mg/mL LPDS and antibiotics. After 12-18, 24, and 48 h, the cells were removed by centrifugation at 500×*g* for 5 min and the supernatants were collected and either used directly in experiments, or stored at −20 °C until use. The control medium was made under identical conditions but without cells.

### Determination of Cellular LDL Degradation

The cellular degradation rate of ^125^I-LDL was used as a measure of LDL uptake [1, 2] and was denoted as “basal LDL degradation rate” of AML cells when measured directly after isolation from blood. Acid soluble cellular degradation products of ^125^I-LDL which are released into the medium were extracted and measured. In brief, 3 × 10^6^ isolated leukemic cells (1 × 10^6^ cells for cell lines) were incubated with 25 μg of ^125^I-LDL for 4 h in 35 × 10 mm tissue culture dishes (Costar Corporation, Cambridge, MA, USA) at 37 °C in 1 mL of RPMI 1640 medium supplemented with 5 mg/mL LPDS and antibiotics (100 IU penicillin + 100 μg streptomycin/mL). For isolated AML cells, incubations were also performed in the absence and presence of 500 μg/mL of unlabelled LDL in order to determine the cellular high affinity degradation rate directly after isolation from blood (here denoted basal high affinity degradation rate) as described previously [1, 2]. After incubation, the cells were spun down and equal volume of cell free medium and ice cold 20% trichloroacetic acid was mixed, and the precipitate was removed by centrifugation. To 1 mL supernatant, 10 µL of 40% KI and 50 µL of 30% H_2_O_2_ were added and mixed. After 5 min the mixture was extracted with 2 mL of chloroform to remove free iodine. The formation of noniodide TCA soluble radioactivity in the aqueous fraction was determined by a gamma counter. The cellular LDL degradation rate was expressed in ng LDL degraded/h/10^6^ cells. For stimulatory experiments (isolated leukemic cells and cell lines) we only determined the total cellular degradation of ^125^I-LDL after adding 25 μg/mL of ^125^I-LDL. Previous studies have shown that the high affinity degradation rate component was >90% of the total degradation rate [1, 2, 28].

### Incubations

Cells (3 million mononuclear blood cells or 1 million cells for cell lines) were preincubated in 1 mL of conditioned LPDS medium or control LPDS medium at 37 °C in 35 × 10 mm tissue culture dishes for the indicated time periods. In some experiments cytokines or neutralizing antibodies were added as indicated to the medium. Thereafter ^125^I-LDL was added to the dishes and the degradation of ^125^I-LDL was determined after 4 h of incubation as described above. Determinations of cellular degradation of LDL in AML cells were performed on freshly isolated cells from blood (denoted basal LDL degradation rate) and following culture in conditioned and control medium for 24 h.

Mononuclear blood cells from AML patients were also incubated in autoconditioned medium. Conditioned medium was obtained as described above. Simultaneously 60 × 10^6^ AML cells were kept at 4 °C in 75-cm^2^ flasks in 20 mL 5 mg/mL LPDS medium to await preparation of autoconditioned medium. The next day, the cells that had been kept at 4 °C, were centrifuged, and diluted to 3 × 10^6^ cells/mL in autoconditioned medium, or control medium. After 24 h at 37 °C the degradation rate of ^125^I-LDL was determined. The stimulation of LDL degradation obtained by conditioned medium compared with control medium was denoted autostimulation.

### Antibodies

Affinity purified rabbit anti-human LDL receptor IgG antibody was purchased from RDI Division of Fitzgerald Industries Intl, Concord, MA, USA and stored at −20 °C. Rabbit IgG isotype control antibody was purchased from abcam, Cambridge, UK and stored at +4 °C.

### Cytokines

Recombinant human IL-1-β, IL-2, IL-4, IL-6, IL-8, IL-9, IL-18, OSM, IFN-γ, and TNF-α (Larodan Fine Chemicals, Malmo, Sweden), and monoclonal anti-human IL-2, anti-human IL-4, anti-human IL-6, and anti-GM-CSF neutralizing antibodies (R&D Systems, Europe, UK), were reconstituted according to the instructions from the suppliers and stored in aliquots at −20 °C.

### Measurements of Cytokines and Growth Factors in Conditioned Media

Commercial enzyme-linked immunosorbent assay (ELISA) kits for the quantitation of cytokines (IL-1β, IL-2, IL-4, IL-6, IL-8, IL-18, IFN-γ and TNF-α) and growth factors (VEGF, HGF and bFGF) in AML conditioned media were employed (R&D systems, Minneapolis, MN, USA). All ELISA kits were used according to the instruction manuals and all measurements were performed in duplicate. The lower quantification limits of each ELISA kit were 1 pg/mL for IL-1β, 0.8 pg/mL for IL-2, 2 pg/mL for IL-4, 10 pg/mL for IL-6, 15 pg/mL for IL-8, 25 pg/mL for IL-18, 5 pg/mL for IFN-γ, 15 pg/mL for TNF-α, 9 pg/mL for VEGF, 40 pg/mL for HGF and 3 pg/mL for bFGF.

### Statistical Analysis

All data are expressed as means and standard deviations of three samples if not othervise stated. Regression lines were calculated according to the method of least squares. The statistical significance of differential findings between experimental groups and controls was determined by Student’s *t* test and was considered significant if two-tailed *P* values were < 0.05.

## Results

### Effect of Conditioned Medium on LDL Degradation in Leukemic Cell Lines

**Fig. 1:**
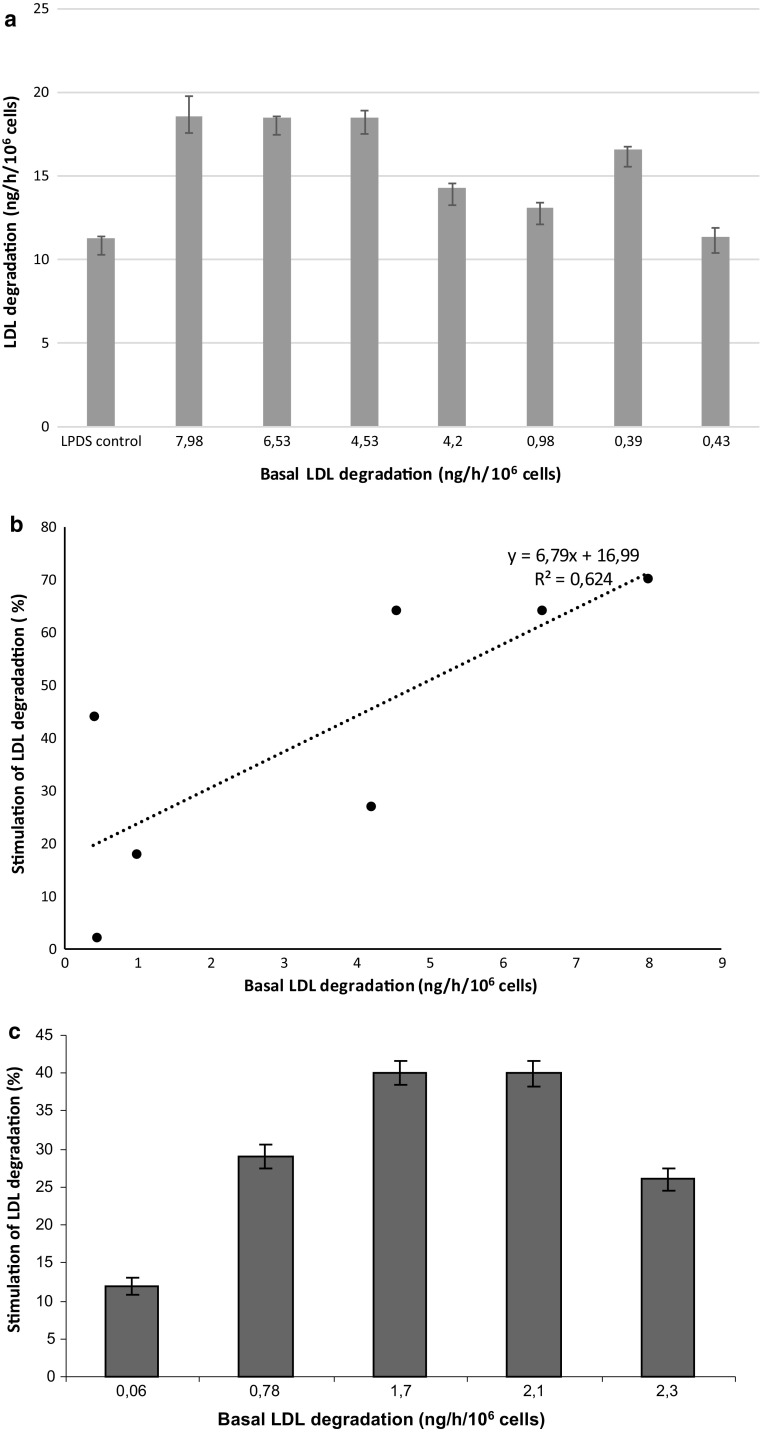
Effect of AML cell-conditioned media on the LDL degradation rate in leukemic cell lines. **a** KG1 cells were preincubated at 37 °C for 5 h with seven conditioned media prepared from AML cells with the given basal LDL degradation rates or non-conditioned control medium (LPDS control). Subsequently cellular LDL degradation rates were determined during a standard 4 h incubation with ^125^I-LDL. All degradation values, compared with control, *p* < 0.01, except for basal degradation 0.43 ng/h/10^6^ cells, NS. Experiments were carried out in triplicate and the mean and standard deviation values are presented in the figure. **b** Relation between the stimulation of LDL degradation in KG1 cells and the basal high affinity degradation rates of AML cells of the conditioned media. Basal LDL degradation value for LPDS medium was set to 0% stimulation. **c** Effect of conditioned media on the stimulation of the LDL degradation rate in HL60 cells. HL60 cells were preincubated at 37 °C for 24 h with 5 different conditioned media prepared from AML cells with basal LDL degradation rates of 0.06, 0.78, 1.7, 2.1, and 2.3 ng/h/10^6^ cells or non-conditioned control medium. Subsequently cellular LDL degradation rates were determined during a standard 4 h incubation with ^125^I-LDL. The LDL degradation rates obtained from conditioned media were compared with that obtained from control LPDS media and the stimulation of LDL degradation was expressed as percentage of control (value for LPDS medium was set to 0% stimulation). Experiments were done in triplicate and the mean and standard deviation values are presented in the figure

We first investigated whether conditioned media from AML cells stimulated LDL degradation in the leukemic cell line KG1. The effects of conditioned media obtained from 7 patients with AML on LDL degradation in KG1 cells, are shown in Fig. 1a. The basal high affinity degradation rates for the AML cells ranged from 0.39 to 7.98 ng/h/10^6^ cells (as a reference, the mean high affinity degradation rate of ^125^I-LDL was 0.40 ng/h/10^6^ cells, range 0.30–0.60 for normal mononuclear blood cells directly after isolation from blood [2]). Following a preincubation with conditioned medium for 5 h, all but one medium stimulated the degradation rate in KG1 cells with maximal stimulation obtained from the conditioned media from AML cells with high basal high affinity degradation rates (Fig. 1a). There was a significant correlation (*R* ^2^ = 0.62, *P* = 0.034) between the basal high affinity degradation rate of the AML cells and the stimulation of degradation in in the KG1 cells by the conditioned media (Fig. 1b).

The leukemic cell line, HL60, also responded to AML cell conditioned media. Five other conditioned media prepared from AML cells with basal high affinity LDL degradation rates of 0.06, 0.78, 1.7, 2.1 and 2.3 ng/h/10^6^ cells were tested and showed variable stimulation of LDL degradation (maximal 40%, Fig. 1c).

### Effect of Conditioned Medium on LDL Degradation in Isolated AML Cells

**Fig. 2:**
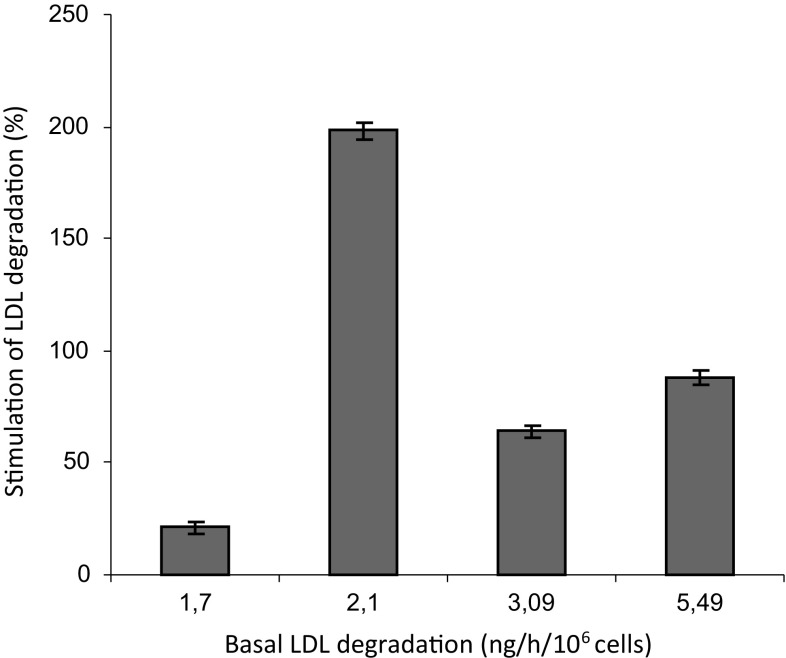
Effect of conditioned media on the LDL degradation rate in isolated AML cells Freshly isolated mononuclear cells from an AML patient with a basal LDL degradation rate of 0.25 ng/h/10^6^ cells were preincubated at 37 °C for 24 h with 4 different conditioned media prepared from AML cells with basal LDL degradation rates of 1.7, 2.1, 3.09, 5.49 ng/h/10^6^ cells or non-conditioned control medium. Subsequently cellular LDL degradation rates were determined during a standard 4 h incubation with ^125^I-LDL. The LDL degradations obtained from conditioned media were compared with that obtained from control LPDS media and the stimulation of LDL degradation was expressed as percentage of control (value for LPDS medium was set to 0% stimulation). Experiments were done in triplicate and the mean and standard deviation values are presented in the figure

We next studied whether the four out of five conditioned media (all from AML cell samples with basal degradation rates > above 1 ng/h/10^6^ cells) that stimulated the LDL degradation rate in KG1 cells also stimulated the degradation rate in AML cells. A clear stimulation of LDL degradation in AML cells with a low basal high affinity degradation rate (0.25 ng/h/10^6^ cells) was observed by the conditioned media (Fig. 2).

### Autostimulation of LDL Degradation

**Fig. 3:**
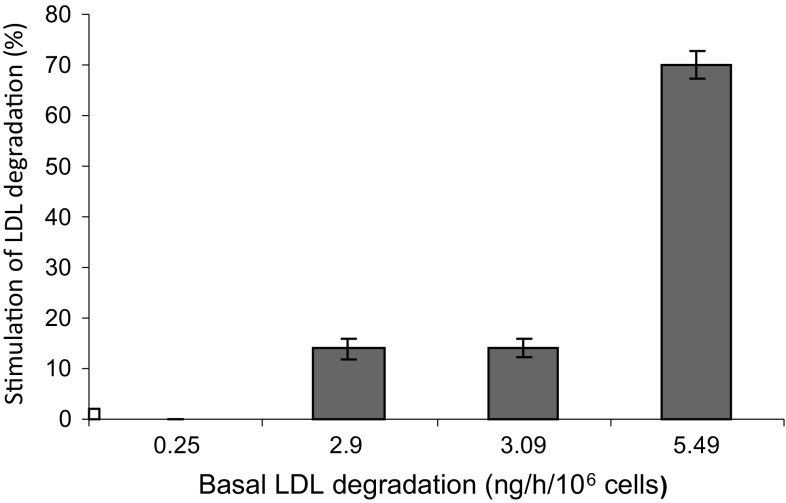
Autostimulation of ^125^I-LDL degradation rate in AML cells. Mononuclear blood cells isolated from four AML patients (basal LDL degradation rates of 0.25, 2.9, 3.09, 5.49 ng/h/10^6^ cells respectively) were preincubated for 4 h with conditioned media prepared from the same cells or non-conditioned control medium. The ^125^I-LDL degradation rates obtained from conditioned media were compared with control LPDS media and the stimulation was expressed as percentage of control (value for LPDS medium was set to 0% stimulation). Experiments were done in triplicate and the mean and standard deviation values are presented in the figure

Our next goal was to study whether conditioned media from isolated AML cells also stimulated the degradation rate in the AML cells that were the origin of the conditioned medium. To perform this autostimulation experiment we incubated freshly isolated leukemic cells from 4 AML patients for 4 h with their conditioned media. The strongest autostimulatory effect (70%) was obtained from conditioned medium from AML cells with the highest basal ^125^I-LDL degradation rate (5.49 ng/h/10^6^ cells) (Fig. 3) while conditioned media from AML cells with a low basal degradation rate did not autostimulate the degradation at all.

### Effect of a Neutralizing Antibody to the LDL Receptor on the LDL Degradation Rate by AML Cells

**Fig. 4:**
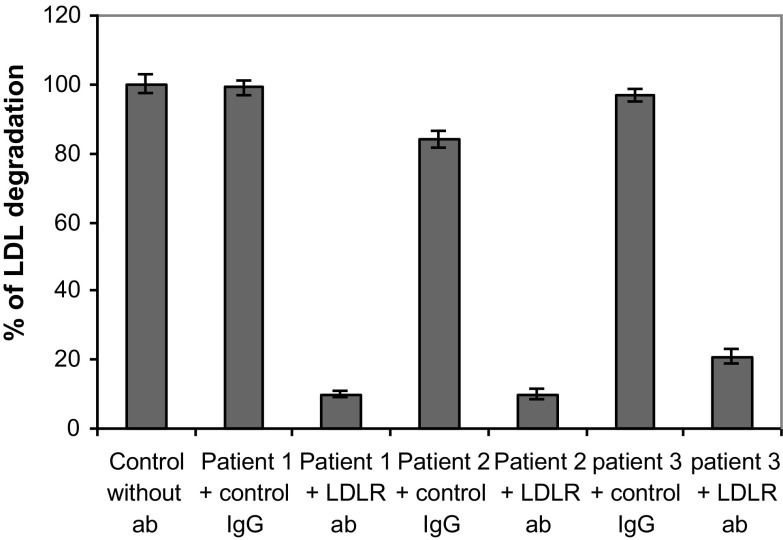
Inhibition of LDL degradation with anti-LDL receptor antibody and control IgG antibodies. Mononuclear blood cells isolated from 3 AML patients were incubated in LPDS media with rabbit IgG antibody or LDL receptor antibody at concentration 10 µg/mL for 30 min prior to incubation with ^125^I-LDL for 4 h. Cells were also incubated in LPDS media under the same condition without any antibody and used as control. The degradation in the control medium without any antibody was expressed as 100% and compared with LDL receptor antibody or control IgG antibody treated samples. Experiments were done in triplicate and the mean and standard deviation values were presented in the figure

In order to obtain support for the “classical” LDL receptor being involved in the elevated uptake and degradation of LDL in leukemic cells we incubated isolated mononuclear blood cells from 3 AML patients with a neutralizing antibody to the LDL receptor. We preincubated the AML cells with an anti-LDL receptor specific rabbit IgG antibody or a control rabbit IgG antibody with no relevant specificity to LDL receptor at 10 µM concentration for 30 min. Thereafter ^125^I-LDL was added and the incubation continued for 4 more hours. We found 70–90% inhibition of ^125^I-LDL degradation in the presence of the specific anti-LDL receptor antibody while the inhibition with the control antibody was only 1–17% (Fig. 4).

### Cytokine Concentration in Conditioned Media

**Fig. 5:**
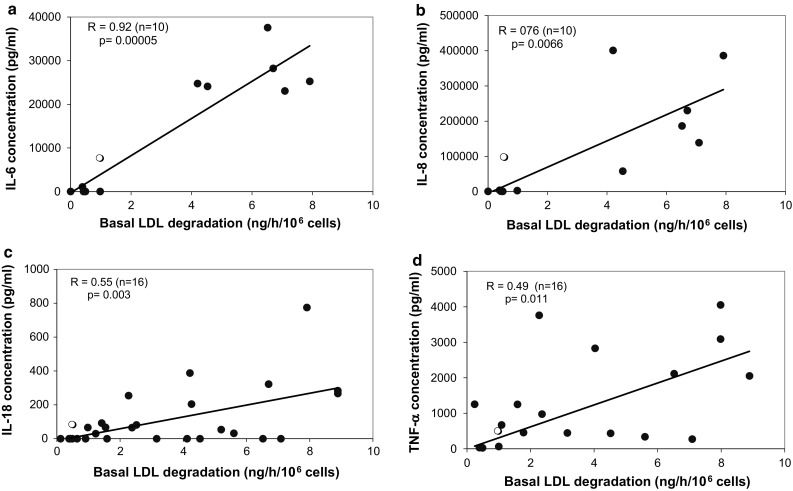
Relationship between the concentration of cytokines in conditioned media and the basal ^125^I-LDL degradation rate in AML cells. **a** IL-6, **b** IL-8, **c** IL-18 and **d** TNF-α. An *open circle* shows the concentration of the respective cytokine in the conditioned media produced by mononuclear blood cells from a healthy subject

In order to find support for the presence of putative stimulating factors we measured the concentration of various cytokines (IL-1β, IL-2, IL-4, IL-6, IL-8, IL-18, IFN-γ, and TNF-α) in conditioned media from AML cells. The correlations between the concentrations of IL-6 (*n* = 10), IL-8 (*n* = 10) IL-18 (*n* = 16), and TNF-α (*n* = 16) in the conditioned and the basal LDL degradation rates are shown in Fig. 5a–d with *R* values of 0.92, 0.76, 0.55, and 0.49 (*p* = 0.0005, 0.0066, 0.003 and 0.011 respectively). For IL-18 and TNF-α several cases with high basal degradation rates had very low or undetectable levels of cytokines and the correlations are weak although significant (*R* ^2^ 0.30 and 0.24 respectively). Concentrations of IL-2, IL-4, and IFN-γ were very low (<12.6 pg/mL) and showed no correlation with basal LDL-degradation rates. The concentration of IL-1β was high in conditioned media from some cases (<24,200 pg/mL) but showed no significant correlation with basal degradation rates overall.

### Effects of Recombinant Cytokines on the LDL Degradation Rate in Isolated AML Cells, HL60, and KG1 Cell Line

**Fig. 6:**
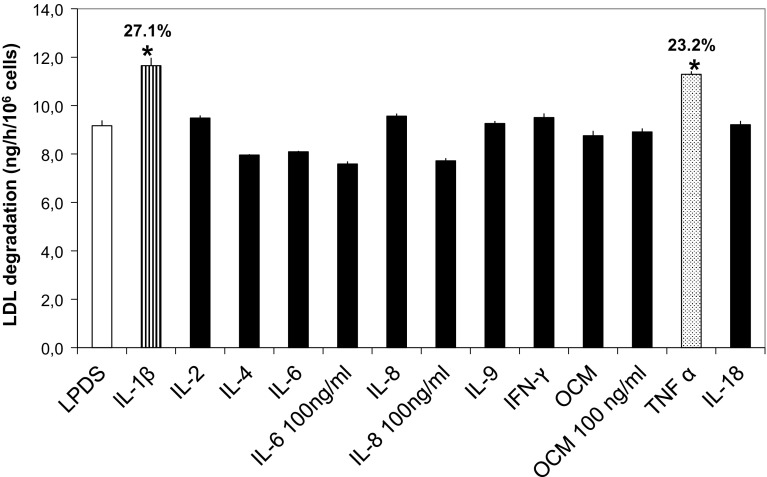
Effect of addition of recombinant cytokines on ^125^I-LDL degradation rate by isolated leukemic cells. Isolated mononuclear blood cells from a subject with AML were preincubated for 20 h with recombinant cytokines (medium concentration 10 ng/mL if not otherwise indicated) whereafter the ^125^I-LDL degradation rate was determined. LDL basal degradation rate (directly after isolation from blood) was: 0.4 ng/h/10^6^ cells. Mean and SD of three incubations. *p < 0.01 compared with LPDS control

We next tested the stimulatory effect of adding 10 recombinant cytokines, IL-1β, IL-2, IL-4, IL-6, IL-8, IL-9, IL-18, OCM, IFN-γ, and TNF-α at 10 ng/mL, IL-8 at 100 ng/mL, and OSM at 50 ng/mL, concentrations that are in the range or exceed the published ED_50s_ of these cytokines. The stimulatory effect of the cytokines on LDL degradation in AML cells with a basal degradation rate of 0.4 ng/h/10^6^ cells is shown in Fig. 6.

There was a weak stimulation by IL-1β and a weak stimulation by TNF-α. No effects were observed by the other cytokines. TNF-α also stimulated (25–50%) LDL degradation in HL60 and KG1 cells after 20 h incubation. The stimulation was concentration dependent, reaching a plateau at 10–20 ng/mL (data not shown). We further observed a 23 and 96% stimulatory effect of LDL degradation by TNF-α in leukemic cells isolated from 2 additional AML patients.

Although the addition of recombinant IL-6 and IL-8 did not stimulate LDL degradation in the in AML cells, we tested the direct addition of an IL-6 neutralizing antibody to AML cells from 2 patients with basal LDL degradation rates of 3.2 and 1.7 ng/h/10^6^ cells. Unexpectedly we found a 50% inhibition of LDL degradation rate in the cells from the first patient with the highest degradation rate while no inhibition at all was observed in the second patient (data not shown).

### Effect of Recombinant Cytokines and Neutralizing Antibodies on LDL Degradation Rate in Isolated Mononuclear Blood Cells from Healthy Subjects

**Fig. 7:**
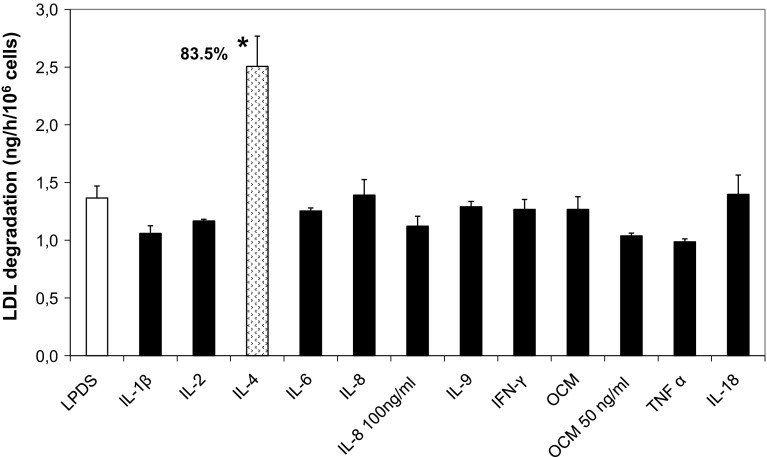
Effect of addition of recombinant cytokines on ^125^I-LDL degradation rate by normal mononuclear blood cells. Normal mononuclear blood cells from a healthy subject were preincubated for 20 h with recombinant cytokines (medium concentration 10 ng/mL if not otherwise indicated) whereafter the ^125^I-LDL degradation rate was determined. Mean and SD of three incubations. *p < 0.01 compared with LPDS control

In order to obtain further support for the involvement of cytokines in LDL uptake stimulation, we also studied the direct effect of adding ten recombinant human cytokines on the LDL degradation rate in mononuclear blood cells from healthy individuals. We found that only IL-4 had a stimulatory effects on ^125^I-LDL degradation in mononuclear blood cells from a healthy subject after 20 h of incubation (Fig. 7). In mononuclear blood cells from another subject, the stimulatory effect was 155% with IL-4 and a slight effect was observed with IL-6 (30%). However, an antibody against IL-4 failed to counteract the stimulation of LDL degradation by AML conditioned media in normal MNC. The same observation was found with an anti-IL-6 antibody (data not shown).

### Growth Factor Concentration in AML-Cell Conditioned Media

Despite the high secretion of IL-6 and IL-8 in conditioned media by AML cells with a high basal LDL degradation rate, the addition of IL-6 and IL-8 failed to stimulate LDL degradation in leukemic cells. We therefore hypothesized that this could be the result of signalling pathways involving growth factors such as VEGF, HGF or FGF and measured the concentration of these factors in conditioned media. The concentration of HGF and FGF did not correlate with LDL basal degradation rate (*R* = 0.053 and 0.019, *n* = 25 and 26 respectively). A weak correlation (*R* = 0.55; *p* = 0.046, *n* = 16) was found between VEGF concentration in conditioned media and the basal LDL degradation rate in the corresponding AML cell samples (not shown). The addition of rhVEGF to AML cells did not stimulate LDL degradation rate (data not shown).

## Discussion

The biological significance of the elevated LDL uptake and hence increased cholesterol supply to AML cells is unknown. We showed earlier that multidrug resistant leukemic K562 cell lines had a 2–10 times elevated uptake of LDL, as well as a significantly higher content of esterified cholesterol when compared with the drug sensitive parental cell lines [29]. It has been proposed that, in AML cells in particular, cholesterol might serve as a protective agent against cellular damage induced by both radiation and chemotherapy [30, 31]. It is therefore of interest to understand the mechanism underlying the elevated LDL uptake in AML cells.

To increase our understanding regarding the mechanism of LDL uptake in AML cells we carried out experiments by incubating leukemic cells with conditioned media derived from AML cells. Our results showed that conditioned media from AML cells with basal high affinity degradation rates above 1 ng/h/10^6^ stimulated LDL degradation in KG1 and HL60 cell lines, and in AML cells, and that the stimulation correlated with the basal high affinity LDL degradation rates of the cells.

Stimulation of LDL degradation has been found to be associated with the secretion of cytokines and growth factors. Earlier studies have reported that oncostatin M, interleukin 6 (IL-6), interleukin 1 (IL-1), transforming growth factor-beta (TGF-β) 1, and tumor necrosis factor (TNF) have been detected in the conditioned media prepared from human macrophages and shown to stimulate LDL uptake and degradation in a human hepatoma cell line [32, 33]. Modulation of LDL receptor transcription by the cytokines appears to occur through different mechanisms. Oncostatin M and IL-6 have been shown to activate the transcription of LDL receptor in HepG2 cells through a sterol independent mechanism [34–36]. Similarly TNF-α, TGF-β, platelet derived growth factor, and IL-1β have been reported to increase LDL receptor transcription in human mesangial cells even in the presence of high levels of cholesterol [37]. In contrast TNF-α and IL-1β are capable of increasing LDL receptor transcription in HepG2 cells only when cells are deprived of sterol, suggesting a sterol dependent mechanism [19]. Thus it appears that LDL receptor transcription responds differently to cytokines in different types of cells.

We could show that the basal LDL degradation rate in freshly isolated AML cells correlated with the secretion of IL-6 and IL-8 and that weak correlations exist with the secretion of IL-18 and TNF-α. A study aiming to characterize cytokine involvement in the pathophysiology of AML showed that IL-6, TNF-α, and IL-1β genes are constitutively expressed in 25, 35, and 31% of AML samples respectively and is associated with the release of the corresponding proteins into AML cell culture media [38]. The *in vitro* secretion of IL-6 and IL-1β by leukemic cells from AML patients was found to be specific for AML subgroups with myelomonocytic (FAB-M4) and monocytic (FAB-M5) differentiation. Similar findings were reported for serum levels of IL-8 in AML patients [39–41]. IL-6 mRNA expression is common in AML cell samples (21%) whereas the IL-6 receptor (IL-6R) is expressed in almost all cases of AML [42]. Serum IL-6 levels were found abnormally elevated in CML in blast crisis and correlated significantly with peripheral white blood cell counts and bone marrow blast counts [43]. IL-6 has been shown to increase the transcription of the LDL receptor gene in HepG2 cells via activation of the sterol-responsive elements in the gene´s promoter. This effect was shown to be independent of the cellular sterol content [36]. Cimino and coauthors found serum IL-2, soluble IL-2R, and TNF-α levels significantly increased in AML patients and found a positive correlation between TNF-α and sIL-2R in the M4-M5 FAB subgroups [44].

We have previously shown that FAB subgroups M4 and M5 are characterized by the highest LDL degradation rate compared to FAB-M1-3 and acute lymphoblastic leukemia [2] which is in agreement with reports showing that TNF-α, IL-6, IL-8, and IL-18 are over-expressed and secreted in these subgroups. The correlations between basal LDL degradation rate in freshly isolated AML cells and their secretion of IL-6, IL-8 TNF-α and IL-18 indicate the involvement of these cytokines in the mechanism of elevated LDL uptake in AML cells. Moreover, LDL degradation was inhibited by 50% with the addition of a neutralizing antibody against IL-6 in the cells from an AML patient with elevated LDL degradation suggesting that IL-6 could be involved in increased LDL uptake process.

A slight stimulation of LDL degradation observed in leukemic cell lines and in AML cells after addition of recombinant TNF-α also indicates the possibility that TNF-α could be involved in the elevated LDL uptake in AML cells. However it does not seem that the secreted cytokines studied here are directly responsible for the elevated LDL uptake since, the direct addition of rIL-6 or IL-8 failed to stimulate LDL degradation in AML cells. The lack of a stimulatory effect when adding IL-6 but a pronounced inhibition of degradation by the addition of an IL-6 neutralizing antibody to AML cells with elevated LDL degradation could possibly be explained by a scenario where an autocrine loop with IL-6 can be blocked but the addition of extra IL-6 will not further stimulate an active stimulatory loop.

It is noteworthy that a strong stimulation of LDL degradation rate was observed in normal mononuclear blood cells after addition of r-IL4 while no effect was seen in leukemic cell lines and AML cells. Since IL-4 was not found in AML cell conditioned media other factors than IL-4 must be responsible for the stimulating effect. This observation indicates differences in LDL uptake stimulation between MNC from healthy subjects and AML patients. A correlation between VEGF concentration in conditioned media and a basal LDL degradation rate in AML cells raises the possibility that cytokine secretion in conditioned media could be a secondary event appearing after cell stimulation by growth factors, although addition of rhVEGF to AML cells did not stimulate the LDL degradation rate. It is therefore possible and even likely, that stimulatory LDL degradation factors play in concert in a complex scheme of events.

The role of the LDL receptor in the uptake of excess amounts of LDL by AML cells is somewhat unclear. A study aiming to determine the expression of LDL receptors in AML cells with high basal LDL degradation rate by ligand blot failed to show increased number of LDL receptors in the AML cells [45]. The authors suggested that AML cells might use other lipoprotein receptors to transport LDL into the cells. We here used a different strategy to study whether or not LDL receptors are involved in the transport of excessive amount of LDL into leukemic cells. We blocked the LDL receptor with a receptor specific antibody to prevent LDL receptor mediated internalization of LDL molecules and measured LDL degradation in the presence and absence of the antibody. We found 70–90% inhibition of LDL degradation when the LDL receptors are blocked by the antibody, supporting the role of LDL receptor in the transport of excess LDL into the AML cells. An immunoglobulin control, produced in the same animal, had only little effect on LDL degradation. Our observation suggests that AML cells use LDL receptors to fulfil their high intracellular demand of LDL, although we can not judge whether AML cells carry out this function by increasing the number of LDL receptors or as a result of high turnover of the LDL receptors, which remains to be studied further.

Our findings are of potential therapeutic interest for AML. If IL-6 and Il-8 are directly involved in autocrine and paracrine loops that stimulate uptake and degradation of LDL by AML cells then this could be used as a therapeutic target for antibodies to these cytokines. The cholesterol needs of the AML cells could be inhibited and such an approach could also be combined with a statin drug that further would inhibit the cholesterol supply to the cells. It is also possible that the cytokines are involved in mechanisms that stimulate AML cell proliferation. Previous studies have also shown the possibility of using LDL as a drug carrier for cytotoxic drugs to target AML cells with elevated LDL uptake [46]. Artificial lipid nanoparticles mimicking the structure and composition of LDL targeting overexpressed LDL receptors in tumor tissue, are also investigated as drug carriers. Such drug loaded nanoparticles have for example shown promising antitumor effects in preclinical human cancer xenograft mouse model studies [47]. Lipid nanoparticles could also be a way to administer water insoluble drugs.

Hypocholesterolemia is frequent finding in patients with AML [48] as well as in other malignancies [49] and our results are interesting as a possible mechanistic explanation for the hypocholesterolemia. LDL uptake and degradation stimulating factors, excreted by the leukemic cells, may also have effects on the liver which is the organ with a central role in regulation of the cholesterol level in the circulation. It is possible that other malignant cells also secrete similar factors which may influence the cholesterol balance in the circulation. Ueyama *et al*. could for example show that culture medium from gallbladder cancer cells, isolated from ascitic fluid from a patient that developed hypocholesterolemia after onset of the disease, stimulated LDL receptor activity in cultured human skin fibroblasts [13].

In this study we could not provide direct evidence for a specific factor secreted by the AML cells that is responsible for the stimulation of LDL degradation. We have, however, shown that (1) AML cells secrete factors that stimulate other cells as well as autostimulate the AML cells themselves (2) The stimulation by AML cell conditioned medium correlates with the basal high affinity degradation rate in the AML cells directly after isolation from blood, and (3) The concentrations of in particular IL-6 and IL-8 in the conditioned media correlated with the basal LDL degradation rates. Our data further support that the elevated LDL uptake in AML cell is accomplished through LDL receptors. We suggest that the uptake process could be stimulated by a complex mechanism involving several cytokines. Further studies are needed to characterize the responsible factors.

## Abbreviations

AML: Acute myelogenous leukemia
HGF: Hepatocyte growth factor
bFGF: Basic fibroblast growth factor
FAB: French-American-British
GM-CSF: Granulocyte macrophage colony stimulating factor
LPDS: Human lipoprotein deficient serum
LDL: Low density lipoprotein
MNC: Mononuclear cells
OSM: Oncostatin M
VEGF: Vascular endothelial growth factor
